# Declining Myocarditis Mortality in the United States and the Impact of the COVID-19 Pandemic

**DOI:** 10.3390/jcm14145116

**Published:** 2025-07-18

**Authors:** Ali Bin Abdul Jabbar, Daniyal Ali Khan, John Osborne, William Thomson, Ameya Chinawalkar, Mason Klisares, Kyle Gilkeson, Ahmed Aboeata

**Affiliations:** 1 Department of Medicine, Division of Internal Medicine, Creighton University School of Medicine, 7710 Mercy Road, Suite 301, Omaha, NE 68124-2368, USAmasonklisares@creighton.edu (M.K.);; 2 Aga Khan University Medical College, Karachi 74800, Pakistan; daniyalali.khan23@alumni.aku.edu; 3 Department of Medicine, Division of Cardiovascular Diseases, Creighton University School of Medicine, Omaha, NE 68124-2368, USA

**Keywords:** myocarditis, mortality, United States, trends, COVID-19 pandemic, disparities

## Abstract

**Background:** Myocarditis is associated with increased mortality due to complications such as cardiogenic shock and arrhythmia. Trends of myocarditis-related mortality in the United States, along with demographic and regional disparities and changes during the COVID-19 pandemic, are unknown. **Methods:** We used the Centers for Disease Control and Prevention Wide-Ranging Online Data for Epidemiologic Research (CDC WONDER) database to extract data for myocarditis deaths from 1999 to 2023. The Joinpoint Regression Program was used to analyze long-term trends in mortality, and R Studio (version 4.4.1) was used to calculate expected and excess mortality for 2020 to 2023. **Results:** There were 33,016 myocarditis-related deaths from 1999 to 2023. The age-adjusted mortality rate (AAMR) of myocarditis deaths decreased by 46.08% from 7.40 (95% CI: 7.04–7.76) in 1999 to 3.99 (95% CI: 3.74–4.23) in 2019, with an APC of −2.59 (95% CI: −2.97 to −2.24). From 2019 to 2021, the AAMR increased by 46.62% to 5.85 (95% CI: 5.56–6.14) by 2021 (2019–2021 APC 22.3%*), reversing the gains of the previous two decades. By 2023, the AAMR recovered to 4.33 (95% CI: 4.09 to 4.58), though mortality was still higher than expected from pre-pandemic trends. From 2020 to 2023, there were 40.12% more deaths than expected, with 54.94% higher mortality in 2021. Briefly, 70.33% of excess myocarditis-related deaths also had COVID-19, with a peak of 76.15% of excess myocarditis deaths in 2021 being reported as involving COVID-19 infection. Significant disparities in mortality trends persisted, with males, NH Black or African Americans, and the elderly having higher mortality rates. **Conclusions:** Myocarditis mortality decreased in the United States from 1999 to 2019 but significantly increased during the COVID-19 pandemic years 2020 and 2021. At the height of the pandemic, COVID-19 infection contributed to almost three-quarters of excess myocarditis mortality. Significant disparities in myocarditis mortality persisted from 1999 to 2023.

## 1. Introduction

Myocarditis, characterized by inflammation of the myocardium, has a vast array of etiologies ranging from idiopathic to infectious, medication-induced, and autoimmune causes. Myocarditis has also been seen as a rare complication of COVID-19 infection, and there has also been an increased risk of myocarditis after mRNA COVID-19 vaccinations, though most cases resolve without severe complications [1,2]. The clinical spectrum of myocarditis varies widely, from asymptomatic cases or only mild illness to severe presentations involving fulminant heart failure, cardiogenic shock, and sudden cardiac death [1,2]. In 2021, there were approximately 1.3 million cases of myocarditis, which contributed to around 31,700 deaths globally [3]. Incidence and deaths attributable to myocarditis have been increasing due to better diagnostic methods and a growing older population [1,2]. In the United States, the incidence of myocarditis-related hospitalizations increased from 95 per million in 2005 to 144 per million in 2014 [4]. The COVID-19 pandemic further increased inpatient encounters related to myocarditis, mainly linked to COVID-19 infection [5]. Most of the current research emphasizes in-hospital mortality rates and has not studied myocarditis deaths that happen outside of hospital settings. Moreover, there is a lack of contemporary data regarding the changes in trends of myocarditis-related mortality rates in the US during the recent COVID-19 pandemic. We aim to analyze trends in myocarditis-related mortality in the United States from 1999 to 2023 to assess for demographic and regional disparities and the impact of the COVID-19 pandemic on long-term trends and related excess mortality from the nationwide database of death certificates in the US.

## 2. Methods

### 2.1. Study Design and Database

The Centers for Disease Control and Prevention Wide-Ranging Online Data for Epidemiologic Research (CDC WONDER) was used to extract data on myocarditis-related mortality in individuals aged ≥15 years. We utilized the Multiple Cause-of-Death Public Use database to identify nationwide death certificates where myocarditis was documented either as the underlying (primary) or as a contributing cause of death [6]. This database has been validated and used to analyze mortality rates for many cardiovascular and non-cardiovascular diseases in the US [7,8,9,10,11]. We obtained data on myocarditis-related deaths and corresponding population estimates from 1999 to 2023 using the International Classification of Diseases (ICD), 10th Revision codes I40.x and I51.4 [12]. Institutional review board approval was not required for this study, as it relied on de-identified, publicly accessible data from the CDC WONDER database.

### 2.2. Study Groups

The analysis incorporated data on biological sex, race/ethnicity, age groups, geographic regions and states, urban-rural status, and place of death. Biological sex was categorized as men and women. Race/ethnicity groups were categorized according to death certificate records as non-Hispanic (NH) White, NH Black or African American (NH-BAA), NH Asian or Pacific Islander (NH-API), and Hispanic or Latino. Geographic regions were defined using US Census Bureau standards and divided into Northeast, Midwest, South, and West. Age groups were divided into young (15–44), middle-aged (45–74), and elderly (≥75).

### 2.3. Statistical Analysis

Myocarditis-related deaths and age-adjusted mortality rates (AAMRs) per 1,000,000 were calculated and used for analysis. AAMRs were standardized to the 2000 US standard population to account for differences in age distribution, facilitating better data comparisons across groups, as outlined in prior studies [13]. To analyze mortality trends, we used the National Cancer Institute’s Joinpoint Regression Program (version 5.3.0) [14]. The primary goal was to assess significant shifts in yearly mortality trends by applying segmented linear models, which provided annual percentage changes (APCs) and corresponding 95% confidence intervals (CIs) for each segment of the AAMR trends. APCs were classified as increasing or decreasing depending on whether the mortality trend over each period significantly deviated from zero, determined by a two-tailed *t*-test. A *p*-value  ≤ 0.05 was considered statistically significant, with significant results marked by an asterisk “*” in the results, tables, and figures.

The years from 2020 to 2023 were separately analyzed for excess myocarditis mortality as a percentage of expected myocarditis-related death, and the proportion of excess myocarditis mortality that involved mention of COVID-19 (ICD 10 code: U07.1) infection on death certificates as an underlying or contributing cause of death was calculated. Excess mortality was calculated by subtracting the expected deaths from the observed deaths.

To determine the expected number of deaths and AAMRs from 2020 to 2023, we employed the autoregressive integrated moving averages (ARIMAs) model using pre-pandemic data (2010 to 2019) to train the model, as previously described [15]. ARIMA models, which incorporate past values (AR terms) and past errors (MA terms), are well-suited for analyzing time-dependent, non-stationary data due to their ability to capture temporal correlations [16]. Model parameters were optimized through the auto.arima() function, selecting the best model based on the Bayesian Information Criterion (BIC) [16,17,18], and all analyses were performed in R (version 4.4.1).

To evaluate stationarity, we applied the Augmented Dickey–Fuller (ADF) test. We then assessed the model’s fit and predictive strength using several performance metrics: Root Mean Squared Error (RMSE), Mean Absolute Error (MAE), Mean Absolute Percentage Error (MAPE), and the first-lag autocorrelation of residuals (ACF1). Additionally, residual diagnostics included the Ljung–Box test to examine autocorrelation across multiple lags, testing whether residuals resembled white noise [18].

We calculated the RMSE on one-step-ahead-fitted values to assess how well the model captured patterns in the historical data. To more rigorously evaluate predictive accuracy, we performed time series cross-validation using the tsCV() function from the forecast package with a 10-step horizon [19], which provided a multi-step RMSE reflecting forecast errors across different points within the training period. This offered a more cautious estimate of forecast reliability.

For both the expected AAMRs and the expected number of deaths, the ADF test indicated non-stationarity, necessitating differencing; consequently, ARIMA (2,1,0) models were selected for each outcome. Model diagnostics and performance metrics are summarized in Table S8. Analysis took place in January 2025, and the RECORD checklist was followed.

## 3. Results

**Figure 1:**
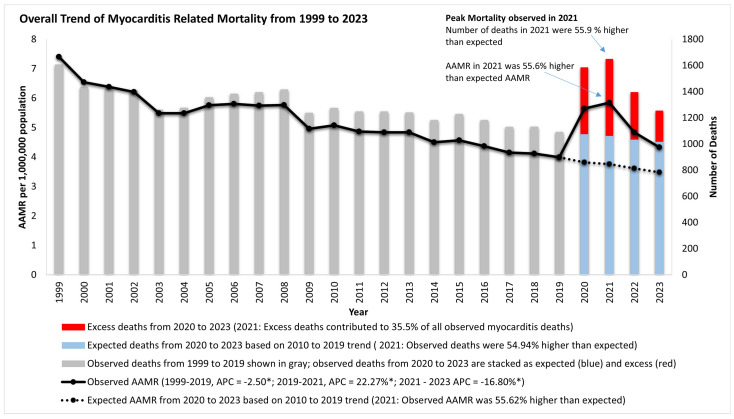
Overall trend of myocarditis mortality in the US from 1999 to 2023. AAMR: age-adjusted mortality rate; APC: annual percentage change (%); AAPC: average annual percentage change (%); PC: percentage change; (* significantly different from 0 at α = 0.05); (* significantly different from 0 at α = 0.05).

There were 33,016 myocarditis-related deaths in the US from 1999 to 2023 (Table S2). In total, 17530 (53.1%) of these were in medical facilities, whereas 15,486 (46.9%) were outside medical facilities (home/hospice facility: 33.7%; nursing homes: 3.6%; other/unknown: 9.6%). The AAMR of myocarditis deaths decreased by 46.1% from 7.40 in 1999 to 3.99 in 2019 with an APC of −2.6*. From 2019 to 2021, the AAMR increased by 46.6% to 5.85 by 2021 (2019–2021 APC 22.3*), reversing the gains of the previous two decades. By 2023, the AAMR had recovered to 4.33 (2021–2023 APC −16.8*) (Figure 1, Table S3).

### 3.1. Impact of the Pandemic, Excess Mortality, and Recovery from 2020 to 2023

The annual number of deaths increased by 51.1% from 1091 in 2019 to a peak of 1648 in 2021, which became the year with the highest number of myocarditis-related deaths from 1999 to 2023 (higher than 1606 deaths in 1999). From 2020 to 2023, there were 1683 (40.1%) more deaths than expected based on the ARIMA model. The year 2021 had the highest excess mortality, with 54.9% higher mortality than predicted. In total, 1184 (70.33%) of excess myocarditis-related deaths between 2020 and 2023 also had COVID-19, with a peak of 76.2% of excess deaths in 2021 reporting COVID-19 infection as a primary or contributing cause of death (Figure 1). By 2023, the AAMR for myocarditis-related mortality was still higher than expected (Table S9), with 43.3% of the excess mortality reporting COVID-19 as an underlying or contributing cause of death in 2023 (Figure 1, Table S6).

A sensitivity analysis focusing on cases where myocarditis was listed as the underlying cause of death revealed a comparable pattern, with AAMRs declining from 3.39 in 1999 to 1.95 in 2023, and showing an APC of −2.2* (Figure S1). COVID-19 was the underlying cause of death for 25.3% of myocarditis-related deaths in 2021 and 18.4% of cumulative myocarditis-related deaths from 2020 to 2023 (Table S7).

### 3.2. Demographic Differences

#### 3.2.1. Sex Stratification

**Figure 2:**
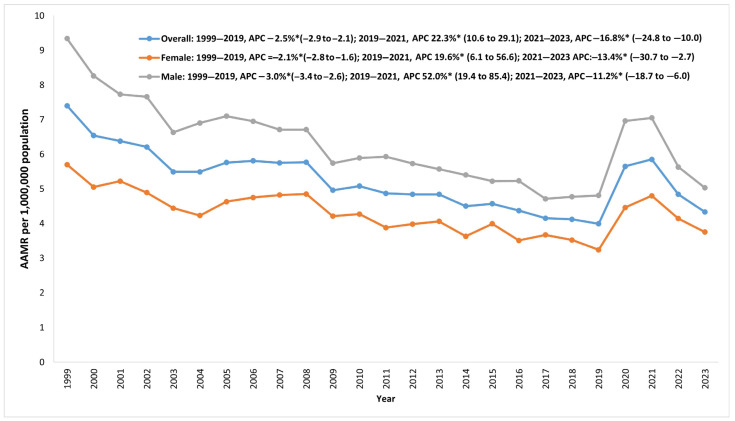
Overall myocarditis AAMRs in the US from 1999 to 2023 and stratified by sex.

Of the myocarditis-related deaths, 18,714 (56.7%) occurred in men and 14,302 (43.3%) occurred in women. Throughout this study, men had higher AAMRs than women but saw a greater reduction in mortality from 1999 to 2019. Men and women saw a similar rise in mortality from 2019 to 2021, increasing by 46.6% and 48.2%, respectively. From 1999 to 2019, AAMR for men decreased by 48.5%, from 9.34 to 4.81, with an APC of −3.0*. This decline was followed by a rise, with the AAMR climbing to 7.05 by 2021 (2019–2021 APC 52.0*), before dropping again to 5.03 by 2023 (2021–2023 APC −11.2%*). The AAMR for women decreased by 43.2%, from 5.7 in 1999 to 3.24 in 2019, with an APC of −2.1*. It increased to 4.8 in 2021 (2019–2021 APC 19.6*), followed by a decrease to 3.75 by 2023 (2021–2023 APC −13.4*) (Table S3, Figure 2).

#### 3.2.2. Race/Ethnicity Stratified

**Figure 3:**
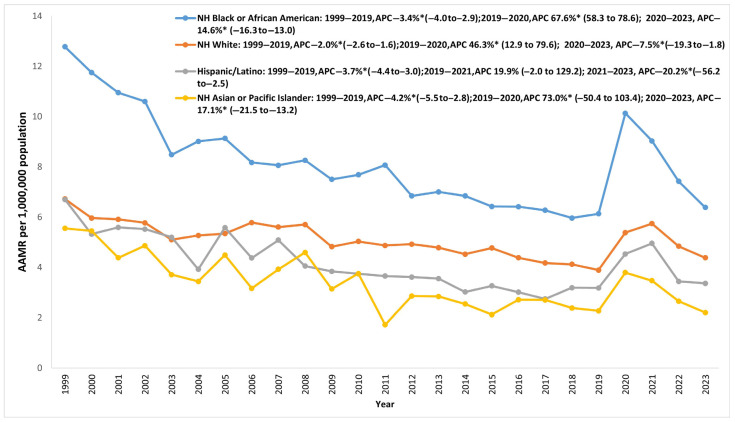
Myocarditis AAMRs in the US from 1999 to 2023, stratified by race/ethnicity. AAMR: age-adjusted mortality rate; APC: annual percentage change (%); AAPC: average annual percentage change (%); PC: percentage change; (* significantly different from 0 at α = 0.05).

Between 1999 and 2019, AAMRs declined across all racial/ethnic groups, but this trend reversed with a rise observed from 2019 through to 2020–2021. NH-BAA had the highest AAMR from 1999 to 2023, which declined by 52% from 12.78 in 1999 to 6.13 in 2019, with an APC of −3.4*. NH White individuals saw a decrease in the AAMR of 42.1%, from 6.72 in 1999 to 3.89 in 2019, with an APC of −2.0*. The AAMR in Hispanic individuals declined by 52.5%, from 6.70 in 1999 to 3.18 in 2019, with an APC of −3.7*, whereas NH-API saw a decrease in the AAMR of 59.1%, from 5.55 in 1999 to 2.27 in 2019, with an APC of –4.2*. During the COVID-19 pandemic, NH-BAA and NH-API had earlier peak mortalities in 2020 and experienced the highest rise in the AAMR of 65.3% and 67.0%, respectively. The AAMRs of Hispanic and NH White people peaked in 2021, increasing by 55.7% and 47.6%, respectively (Table S1, Figure 3).

#### 3.2.3. Age Group Stratification

**Figure 4:**
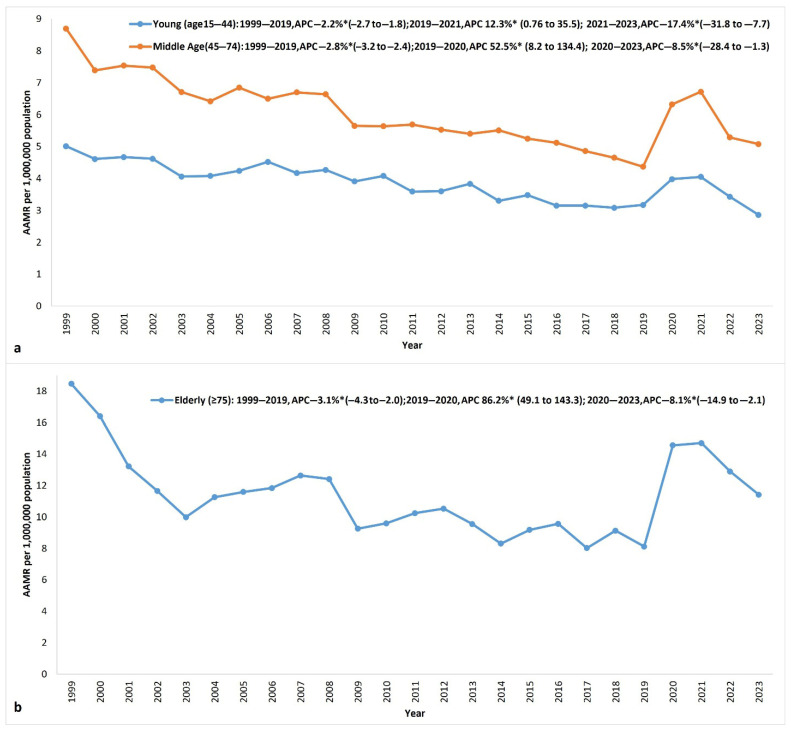
Myocarditis-related AAMRs (95% CI) in the US from 1999 to 2023, stratified by age groups: (**a**) young (15–44-year-old) and middle-aged (45–74-year-old), and (**b**) older (≥75-year-old) groups. AAMR: age-adjusted mortality rate; APC: annual percentage change (%); AAPC: average annual percentage change (%); PC: percentage change; (* significantly different from 0 at α = 0.05).

In total, 15,242 (46.2%) deaths were in middle-aged individuals (45–74), followed by 12,094 (36.6%) young (15–44) and 5680 (17.2%) elderly individuals (≥75 years). Throughout the study period, individuals in the older age group (≥75 years) consistently exhibited the highest AAMRs, with progressively lower rates seen in the middle-aged (45–74) and younger (15–44) groups. From 1999 to 2019, the older age group saw the greatest reduction in AAMR of 56% from 18.46 in 1999 to 8.12 in 2019, with an APC of –3.1*. Younger individuals saw the lowest mortality reduction of 36.7%, from 5.01 in 1999 to 3.17 in 2019, with an APC of −2.2*, and middle-aged individuals saw a decrease of 49.8%, from 8.7 in 1999 to 4.37 in 2019, with an APC of −2.8*. All age groups saw an increase in mortality from 2019 to 2021, with the elderly population experiencing the largest AAMR increase of 81.0%, followed by 53.8% in the middle-aged group and a 27.8% increase in the younger age group (Table S1, Figure 4).

The AAMRs for all demographic and regional subgroups recovered by 2023 to the levels seen in 2019, except for the AAMR of elderly individuals, which remained elevated at 11.4 in 2023 compared to the AAMR of 8.12 in 2019 (Table S1).

### 3.3. Regional Differences

#### 3.3.1. Census Region Stratification

**Figure 5:**
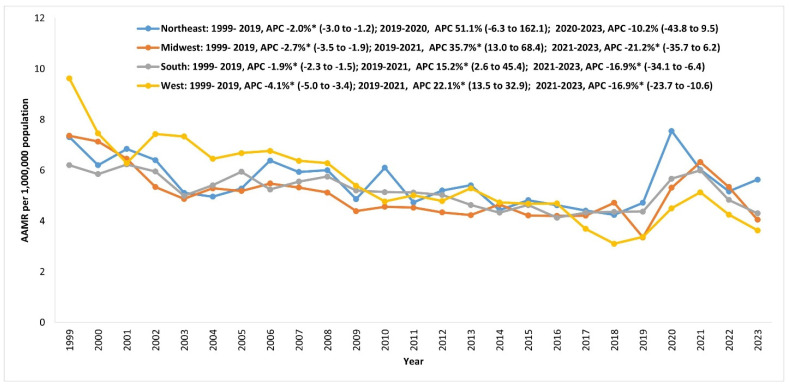
Myocarditis AAMRs (95% CI) stratified by US census regions in the US from 1999 to 2023. AAMR: age-adjusted mortality rate; APC: annual percentage change (%); AAPC: average annual percentage change (%); PC: percentage change; (* significantly different from 0 at α = 0.05).

Most census regions had similar AAMRs from 1999 to 2023. AAMRs declined across all census regions between 1999 and 2019. The West region had the highest AAMR in 1999 but experienced the largest decrease of 65%, from 9.62 in 1999 to 3.37 in 2019 (APC −4.1*), becoming the lowest along with the Midwest (Table S4). The southern region saw the smallest decrease in AAMR of 29.5%, from 6.20 in 1999 to 4.37 in 2019, with an APC of −1.9*. From 2019 to 2021, all regions saw mortality increases, with the Midwest region experiencing the most significant AAMR increase of 88.7%, followed by the West (52.2%) and South (37.1%). The Northeast region had a peak in 2020, earlier than other regions, with the AAMR increasing by 60% from 2019 to 2020 (Table S1, Figure 5).

#### 3.3.2. State-Level Differences

**Figure 6:**
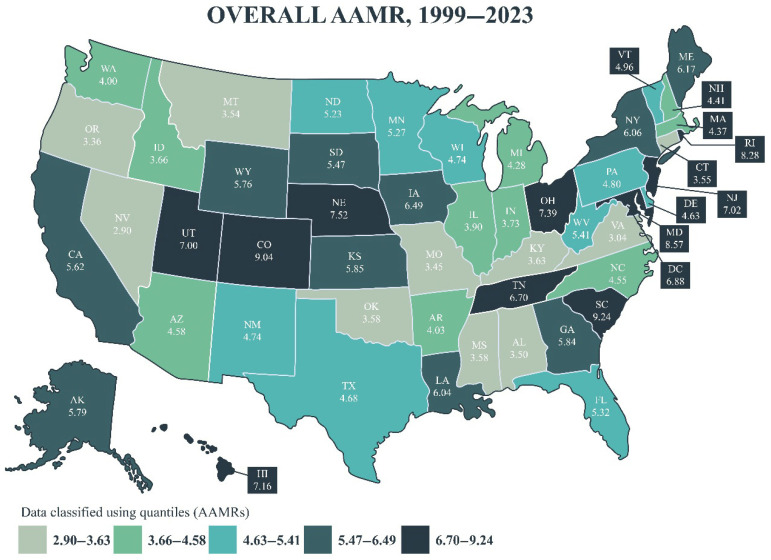
State-level differences in myocarditis AAMRs in the United States from 1999 to 2023; AAMR: age-adjusted mortality rate.

From 1999 to 2023, the highest AAMRs were in South Carolina (9.24), Colorado (9.04), and Maryland (8.57) (Figure 6, Table S5). From 1999–2019 to 2020–2023, Nebraska experienced the largest increase in the AAMR of 5.85 (89.9%), from 6.51 to 12.36, taking the top spot for AAMRs in the 2020–2023 period, while Rhode Island saw the greatest decrease in the AAMR of –3.83 (−42.9%).

## 4. Discussion

Our 25-year analysis of myocarditis-related mortality in the US highlights several interesting findings. Myocarditis-related mortality declined significantly in the US from 1999 to 2019. A significant spike in deaths was noted during the COVID-19 pandemic years of 2020 and 2021, reversing the gains of the prior two decades. This was followed by an appreciable recovery in 2022 and 2023, albeit at higher-than-expected levels based on pre-pandemic trends. COVID-19 infection coincided with almost three-quarters of the excess myocarditis mortality. It was listed as the underlying cause of death in almost 1/4th of all myocarditis-related deaths at the height of the pandemic in 2021, and in 1/5th of cumulative myocarditis-related deaths recorded from 2020 to 2023. Men and NH-BAA individuals had higher AAMRs throughout the study period. Among all age groups, older adults consistently exhibited the highest AAMRs, experienced the largest drop between 1999 and 2019, and saw the most pronounced rise amid the COVID-19 pandemic. The West region had the highest AAMR at the beginning of the study period but saw the greatest decline over time, ultimately reaching levels comparable to those in the Midwest and South, and falling below those of the Northeast.

The decline in myocarditis mortality seen in our study from 1999 to 2019 is consistent with other regional and global declines for the 1990 to 2019 period [3]. This decline in myocarditis-related mortality in the United States and worldwide has been linked to advancements in the timely diagnosis and treatment of myocarditis. Myocarditis has been considered an underdiagnosed condition; historically diagnosed through endomyocardial biopsy, a combination of newer tools like cardiac magnetic resonance imaging (MRI) and high-sensitivity troponin tests is now used for diagnosis [1,2]. Advancements in diagnostic techniques, particularly MRI, have significantly improved the early diagnosis of myocarditis, allowing for timely management and better patient outcomes [20].

Treatment of myocarditis, which involves managing heart failure and arrhythmias, has advanced as well [1,2]. The use of advanced mechanical circulatory support (MCS) devices such as extracorporeal membrane oxygenation (ECMO) and percutaneous ventricular assist devices (VADs) has increased dramatically, acting as a breakthrough for critical support in cases of severe myocarditis complicated by cardiogenic shock, thereby improving survival rates by buying time for the treatment of the underlying etiology of myocarditis and recovery of the myocardium [4,21]. Treatment is also tailored to the underlying cause, including therapies such as antibiotics for bacterial causes and systemic corticosteroids for autoimmune/immune-mediated causes [1,2]. Standardized clinical guidelines issued by leading cardiology organizations, including the American College of Cardiology, have further enhanced treatment approaches [22].

Furthermore, our study is consistent with the existing literature reporting myocarditis as a rare but life-threatening complication of COVID-19 infection. The prevalence of acute myocarditis among hospitalized COVID-19 patients ranged from 1.28 to 4.1 cases per 1000 hospitalizations, with an in-hospital mortality rate of as much as 20.4% to 30% [23,24,25]. It has also been reported to occur without concurrent pneumonia in COVID-19 infection, often presenting with elevated cardiac biomarkers and left ventricular dysfunction, and has been linked to increased adverse events such as cardiac arrest and cardiogenic shock [25]. Aside from novel COVID-19 infection, myocarditis has also been implicated as a rare complication of COVID-19 vaccines, specifically, the mRNA-based vaccine formulations [1,2,26]. However, the elevated risk of myocarditis associated with vaccination has been a tiny fraction of the risk associated with COVID-19 infection, even for higher-risk subgroups such as young male adults [26,27,28]. It is crucial to acknowledge this difference to hypothesize the temporal factors that may explain the trend in myocarditis-related mortality from 2020 to 2023. Myocarditis-related mortality increased sharply in 2020, peaked in 2021, and then declined gradually until 2023. COVID-19 seropositivity was estimated in 16% of the population by September 2020 [29]. The Omicron surge occurred in late 2021 and early 2022, leading to the highest recorded case rates. By November 2022, approximately 97% of the US population had some immunological exposure via infection, vaccination, or both; however, the proportion of the vaccinated population increased from 2021 to 2023 [30,31]. Reinfection with subsequent strains continued after 2022, possibly contributing to the elevated myocarditis-related mortality observed in 2022 and 2023.

Higher rates of myocarditis mortality in men compared to women have been corroborated in prior studies as well [32]. A similar trend continued through the COVID-19 pandemic, with men having higher mortality. A proposed reason for this is that men tended to have a more robust proinflammatory cytokine milieu when infected with COVID-19 compared to women [33]. NH-BAA consistently recorded the highest AAMRs across the entire study timeframe. The disparity recognized in the African American population has been seen across the spectrum of cardiovascular disease, being linked to inequalities in access to care, lower rates of advanced therapies, and a higher prevalence of comorbid conditions such as hypertension, diabetes, and obesity, which can exacerbate the severity of myocarditis and complicate its management. Socioeconomic determinants, including lower income, gaps in insurance coverage, and limited availability or utilization of preventive services, also contribute to poorer overall health and delayed medical intervention [8,34,35]. A study on myocarditis and cardiomyopathy hospitalizations in the pediatric population reported that African Americans had a more severe illness at the time of admission and were less likely to obtain heart transplants, highlighting potential healthcare access disparities [36]. Other hypotheses include genetic predispositions and biological differences; for instance, lower levels of circulating progenitor cells, which are crucial for cardiovascular repair, have been observed in Black patients, potentially leading to poorer outcomes of myocarditis in this group [37]. Elderly individuals tend to have multiple chronic comorbidities that compromise vital organ systems, leading to decreased health reserves and making them more vulnerable [38]. A study in Sweden showed that those aged ≥50 years had 20.8% mortality within a year of diagnosis compared to a 0.9% one-year mortality rate for those aged < 50 years from 2000 to 2014 [39]. Overall, these disparities are likely multifactorial, involving patient-, provider-, and system-level factors. Although investigating these contributors was beyond the scope of our study, our findings underscore the persistent inequities in myocarditis-related health outcomes within the US healthcare system.

All regions had a declining trend in mortality from 1999 to 2019; however, the West region showed the most improvement in AAMR. It is unclear how the West improved more than other regions to end up with a mortality rate similar to that of the South and Midwest and lower than that of the Northeast region, after starting with the highest AAMR compared to the different regions studied. This could be an opportunity for further research to explore. The Northeast region saw the COVID-19 pandemic-related peak in 2020, earlier than another region that saw peak mortality in 2021. This is exemplified by New York and neighboring states, which became the epicenter of the COVID-19 pandemic [40]. One hypothesis to explain this trend is that myocarditis cases were significantly underdiagnosed in rural areas earlier in the study, leading to falsely low AAMR in regions and states at the start of the study that were predominantly rural areas. Improvement in the accessibility of diagnostic resources over the past two decades has led to better capture of myocarditis cases. State-level differences in the increase in AAMRs during COVID-19 are also hypothesized to be partly from the unique demographic mix and geographic spread of the population in each state. During the pandemic, for instance, just over 30% of Louisiana’s population comprised African Americans, but 70.5% of deaths occurred among African Americans, corroborating the racial disparities at a state level. This highlights that relatively predominant minority demographic groups, along with their higher comorbidity burden and social vulnerability of the area, play a role in state-level variability.

Our study included extensive data from the past 25 years and is the only study to include a comprehensive analysis of myocarditis-related mortality in the US through 2023. However, our study has some limitations. The CDC WONDER database is based on death certificate data that uses ICD-10 coding to classify diseases, which introduces the risk of misclassification bias and may also reflect regional variations in reporting practices. This analysis was limited to examining racial disparities among NH White, NH-BAA, NH-API, and Hispanic or Latino populations. NH American Indian or Alaska Native populations could not be included in the analysis due to suppression in most years across subgroups. We acknowledge that further stratified analyses by combined demographic and geographic subgroups could uncover additional disparities; however, such an in-depth exploration exceeds the scope of the present national-trend study. To protect confidentiality, the CDC suppresses counts below 10 in WONDER data, per the data use agreement, and death rates based on fewer than 20 cases are flagged as unreliable. Moreover, due to the ecological nature of the data, a causal relationship cannot be established between COVID-19 and myocarditis for deaths during 2020–2023.

## 5. Conclusions

In the United States, myocarditis-related mortality rates declined from 1999 to 2019, but this progress was notably reversed with a significant rise amid the COVID-19 pandemic years of 2020 and 2021. At the height of the pandemic, nearly three-quarters of the excess myocarditis deaths occurred in patients with COVID-19 infection listed on their death certificates. While mortality has decreased since 2021, it has not yet returned to the level expected based on the pre-pandemic trends. Significant disparities in mortality trends persisted, with males, NH Black or African Americans, and the elderly having higher mortality rates throughout 1999 to 2023.

## Data Availability

All data are available at: https://wonder.cdc.gov/.

## Supplementary Materials

The following supporting information can be downloaded at: https://www.mdpi.com/article/10.3390/jcm14145116/s1, Table S1: Descriptive Summary of trend and disparities in myocarditis-related deaths and AAMR in 1999, 2019, 2020/2021, 2023 and percentage change in AAMR; Table S2: Myocarditis-related annual number of deaths stratified by sex, race, region, age group, and Census region in the United States, 1999–2023; Table S3: Myocarditis-related annual age-adjusted mortality rates per 1000,000 stratified by sex, race, and age in the United States, 1999–2023; Table S4: Myocarditis-related annual age-adjusted mortality rates are stratified by the census region in the United States, 1999–2023; Table S5: Myocarditis-related age-adjusted mortality rate (AAMR) in 1999–2019 and 2020–2023 and change in AAMR at the state level in the United States, 1999–2023; Table S6: Observed and expected myocarditis deaths and contribution of COVID-19 infection to the excess myocarditis deaths; Table S7: Sensitivity analysis of most frequently mentioned underlying (primary) causes of death for all myocarditis-related deaths for cumulative 2020–2023, year 2021 and cumulative 2016–2019 for comparison; Table S8: ARIMA model diagnostics and metrics for expected AAMRs and number of deaths; Table S9: Forecast of expected AAMRs from 2020 to 2023 using the ARIMA model; Figure S1: Sensitivity analysis for Myocarditis-related AAMR from multiple cause-of-death data (all myocarditis-related deaths) and the underlying cause of death data; Figure S2: State-level myocarditis-related AAMR from 1999 to 2019 and 2020 to 2023; Figure S3a: State-level change in Myocarditis-related age-adjusted mortality rates (AAMR) in the United States from 1999–2019 to 2020–2023; Figure S3b: State-level change in Myocarditis-related age-adjusted mortality rates (AAMR) in the United States from 1999–2019 to 2020–2023.
