# Supplementary material for: Declining Myocarditis Mortality in the United States and the Impact of the COVID-19 Pandemic
Source: J Clin Med. 2025 Jul 18;14(14):5116. doi: 10.3390/jcm14145116 (PMC12295393; doi:10.3390/jcm14145116)

Supplement Materials

Table S1. Descriptive Summary of trend and disparities in myocarditis-related deaths and AAMR in 1999, 2019, 2020/2021, 2023 and percentage change in AAMR (AAMR: Age-adjusted mortality rate, NA: not available; *NH Black or African American, NH Asian or Pacific Islander and Northeast region had peak AAMR in 2020 so that was used to calculate percentage increase in AAMR)

	Number of deaths				AAMR (95% CI)				Percentage change in AAMR (%)		
	1999	2019	2021	2023	1999	2019	2021	2023	From 1999 to 2019	From 2019 to 2021	From 2019 to 2023
**Overall Myocarditis Mortality**	1606	1091	1648	1253	7.40 (7.04 to 7.76)	3.99 (3.74 to 4.23)	5.85 (5.56 to 6.14)	4.33 (4.09 to 4.58)	-46.08	46.62	8.52
**Sex stratified**											
Male	936	624	951	700	9.34 (8.73 to 9.95)	4.81 (4.42 to 5.19)	7.05 (6.60 to 7.51)	5.03 (4.65 to 5.42)	-48.50	46.57	4.57
Female	670	467	697	553	5.70 (5.27 to 6.14)	3.24 (2.94 to 3.55)	4.80 (4.43 to 5.16)	3.75 (3.42 to 4.07)	-43.16	48.15	15.74
**Race Stratified**											
NH Black or African American	305	195	294	214	12.78 (11.31 to 14.25)	6.13 (5.26 to 7.01)	10.13 (9.01 to 11.26) *	6.38 (5.51 to 7.25)	-52.03	65.25**	4.08
NH White	1117	701	1059	821	6.72 (6.33 to 7.12)	3.89 (3.59 to 4.20)	5.74 (5.37 to 6.10)	4.38 (4.06 to 4.70)	-42.11	47.56	12.60
Hispanic	129	133	210	151	6.70 (5.40 to 8.01)	3.18 (2.62 to 3.74)	4.95 (4.25 to 5.65)	3.36 (2.80 to 3.91)	-52.54	55.66	5.66
NH Asian or Pacific Islander	38	38	58	41	5.55 (3.77 to 7.88)	2.27 (1.60 to 3.13)	3.79 (2.89 to 4.88) *	2.20 (1.58 to 2.99)	-59.10	66.96**	-3.08
**Census Region**											
Northeast	316	228	297	292	7.31 (6.51 to 8.12)	4.72 (4.08 to 5.35)	7.55 (6.76-8.34) *	5.63 (4.96 to 6.30)	-35.43	59.96**	19.28
Midwest	371	193	367	238	7.36 (6.62 to 8.11)	3.35 (2.86 to 3.84)	6.32 (5.65 to 6.99)	4.05 (3.52 to 4.58)	-54.48	88.66	20.90
South	475	448	641	471	6.20 (5.64 to 6.75)	4.37 (3.95 to 4.78)	5.99 (5.52 to 6.47)	4.30 (3.90 to 4.69)	-29.52	37.07	-1.60
West	444	222	343	252	9.62 (8.72 to 10.51)	3.37 (2.92 to 3.82)	5.13 (4.58 to 5.68)	3.63 (3.17 to 4.09)	-64.97	52.23	7.72
**Age groups (Crude rate)**											
Young (15-44)	618	398	523	374	5.01 (4.62 to 5.41)	3.17 (2.85 to 3.48)	4.05 (3.70 to 4.40)	2.86 (2.57 to 3.15)	-36.73	27.76	-9.78
Middle age (45-74)	687	502	795	599	8.70 (8.05 to 9.35)	4.37 (3.98 to 4.76)	6.72 (6.24 to 7.20)	5.08 (4.66 to 5.50)	-49.77	53.78	16.25
Elderly (≥75)	301	191	330	280	18.46 (16.37 to 20.54)	8.12 (6.96 to 9.27)	14.70 (13.12 to 16.29)	11.42 (10.08 to 12.76)	-56.01	81.03	40.64

Table S2. Myocarditis-related annual number of deaths stratified by sex, race, region, age group, and Census region in the United States, 1999-2023.

Year	Overall	Female	Male	NH Asian or Pacific Islander	NH Black or African American	NH White	Hispanics	Young (15-44)	Middle age (45-74)	Elderly (≥75)	Northeast	Midwest	South	West
**1999**	1,606	670	936	38	305	1,117	129	618	687	301	316	371	475	444
**2000**	1,434	596	838	42	283	993	106	569	593	272	268	362	456	348
**2001**	1,422	619	803	37	274	989	102	574	625	223	298	330	495	299
**2002**	1,406	589	817	40	269	968	116	569	637	200	284	274	482	366
**2003**	1,261	539	722	33	226	865	121	504	584	173	228	253	410	370
**2004**	1,276	522	754	32	242	896	98	503	575	198	222	276	451	327
**2005**	1,356	570	786	45	242	916	143	524	625	207	235	270	506	345
**2006**	1,383	598	785	31	222	992	127	557	611	215	283	292	450	358
**2007**	1,394	623	771	37	224	980	143	513	646	235	273	288	486	347
**2008**	1,415	631	784	51	234	999	112	523	658	234	270	282	513	350
**2009**	1,236	555	681	40	224	840	121	482	578	176	220	239	471	306
**2010**	1,274	574	700	46	224	881	112	504	581	189	281	248	470	275
**2011**	1,247	525	722	22	241	860	118	442	601	204	224	246	481	296
**2012**	1,247	548	699	38	209	874	118	445	582	220	250	236	474	287
**2013**	1,239	556	683	39	214	835	133	473	569	197	255	233	436	315
**2014**	1,181	504	677	37	211	812	113	411	593	177	215	257	417	292
**2015**	1,228	561	667	32	199	859	127	440	589	199	231	239	460	298
**2016**	1,181	508	673	42	202	807	112	398	572	211	221	246	413	301
**2017**	1,129	512	617	43	201	761	111	401	548	180	216	239	434	240
**2018**	1,131	505	626	36	191	754	127	393	530	208	209	271	444	207
**2019**	1,091	467	624	38	195	701	133	398	502	191	228	193	448	222
**2020**	1,584	653	931	61	324	981	186	505	731	348	380	307	599	298
**2021**	1,648	697	951	58	294	1,059	210	523	795	330	297	367	641	343
**2022**	1,394	627	767	46	242	918	158	451	631	312	260	314	527	293
**2023**	1,253	553	700	41	214	821	151	374	599	280	292	238	471	252
**Total**	33,016	14,302	18,714	1,005	5,906	22,478	3,227	12,094	15,242	5,680	6,456	6,871	11,910	7,779
**Percentage of total (%)**	-	43.3	56.7	3.0	17.9	68.1	9.8	36.6	46.2	17.2	19.6	20.8	36.1	23.6

Table S3. Myocarditis-related annual age-adjusted mortality rates per 1000,000 stratified by sex, race, and age in the United States, 1999-2023.

Year	Overall	Female	Male	NH Asian or Pacific Islander	NH Black or African American	NH White	Hispanics	Young (15-44)	Middle Age (45-74)	Elderly (≥75)
**1999**	7.4	5.7	9.34	5.55	12.78	6.72	6.7	5.01	8.7	18.46
**2000**	6.54	5.05	8.26	5.45	11.75	5.96	5.32	4.61	7.39	16.41
**2001**	6.38	5.22	7.73	4.38	10.95	5.91	5.59	4.67	7.54	13.22
**2002**	6.21	4.89	7.66	4.86	10.6	5.77	5.52	4.62	7.48	11.66
**2003**	5.49	4.44	6.63	3.71	8.48	5.1	5.2	4.06	6.71	9.99
**2004**	5.49	4.23	6.9	3.44	9.01	5.27	3.92	4.08	6.42	11.26
**2005**	5.76	4.63	7.1	4.49	9.13	5.34	5.58	4.24	6.85	11.59
**2006**	5.81	4.75	6.95	3.16	8.17	5.78	4.37	4.52	6.5	11.84
**2007**	5.75	4.82	6.71	3.92	8.06	5.6	5.08	4.17	6.7	12.64
**2008**	5.77	4.85	6.71	4.59	8.26	5.7	4.05	4.27	6.64	12.41
**2009**	4.96	4.21	5.74	3.14	7.5	4.82	3.84	3.91	5.65	9.26
**2010**	5.08	4.27	5.89	3.75	7.68	5.03	3.74	4.08	5.64	9.6
**2011**	4.87	3.88	5.93	1.72	8.07	4.87	3.65	3.59	5.69	10.24
**2012**	4.84	3.98	5.73	2.86	6.84	4.92	3.61	3.6	5.53	10.53
**2013**	4.84	4.06	5.57	2.84	7	4.78	3.55	3.83	5.4	9.55
**2014**	4.5	3.63	5.4	2.54	6.84	4.52	3.02	3.3	5.51	8.31
**2015**	4.57	3.99	5.22	2.12	6.42	4.77	3.26	3.48	5.25	9.18
**2016**	4.37	3.51	5.23	2.71	6.41	4.38	3.01	3.15	5.12	9.57
**2017**	4.15	3.67	4.71	2.7	6.27	4.17	2.74	3.15	4.86	8.02
**2018**	4.12	3.52	4.77	2.38	5.96	4.12	3.19	3.08	4.65	9.13
**2019**	3.99	3.24	4.81	2.27	6.13	3.89	3.18	3.17	4.37	8.12
**2020**	5.65	4.46	6.96	3.79	10.13	5.38	4.53	3.98	6.32	14.56
**2021**	5.85	4.8	7.05	3.47	9.03	5.74	4.95	4.05	6.72	14.7
**2022**	4.84	4.14	5.63	2.65	7.42	4.84	3.44	3.43	5.29	12.9
**2023**	4.33	3.75	5.03	2.2	6.38	4.38	3.36	2.86	5.08	11.42
**Number of Joinpoints (Joinpoint years)**	2 (2019, 2021)	2 (2019, 2021)	2 (2019, 2020)	2 (2019, 2020)	2 (2019, 2020)	2 (2019, 2020)	2 (2019, 2021)	2 (2019, 2021)	2 (2019, 2020)	2 (2019, 2020)
**APC-Segment 1 (95% CI)**	-2.5%* (-2.9 to -2.1)	-2.1%* (-2.8 to -1.6)	-3.0%* (-3.4 to -2.6)	-4.2%* (-5.5 to -2.8)	-3.4%* (-4.0 to -2.9)	-2.0%* (-2.6 to -1.6)	-3.7%* (-4.4 to -3.0)	-2.2%* (-2.7 to -1.8)	-2.8%* (-3.2 to -2.4)	-3.1%* (-4.3 to -2.0)
**APC-Segment 2 (95% CI)**	22.3%* (10.6 to 29.1)	19.6%* (6.1 to 56.6)	52.0%* (19.4 to 85.4)	73.0%* (-50.4 to 103.4)	67.6%* (58.3 to 78.6)	46.3%* (12.9 to 79.6)	19.9% (-2.0 to 129.2)	12.3%* (0.76 to 35.5)	52.5%* (8.2 to 134.4)	86.2%* (49.1 to 143.3)
**APC-Segment 3 (95% CI)**	-16.8%* (-24.8 to -10.0)	-13.4%* (-30.7 to -2.7)	-11.2%* (-18.7 to -6.0)	-17.1%* (-21.5 to -13.2)	-14.6%* (-16.3 to -13.0)	-7.5%* (-19.3 to -1.8)	-20.2%* (-56.2 to -2.5)	-17.4%* (-31.8 to -7.7)	-8.5%* (-28.4to -1.3)	-8.1%* (-14.9 to -2.1)

Table S4. Myocarditis-related annual age-adjusted mortality rates are stratified by the census region in the United States, 1999-2023.

Year	Northeast	Midwest	South	West
1999	7.31	7.36	6.2	9.62
2000	6.2	7.13	5.85	7.45
2001	6.84	6.45	6.23	6.28
2002	6.4	5.34	5.95	7.43
2003	5.11	4.87	4.99	7.33
2004	4.96	5.29	5.41	6.45
2005	5.28	5.18	5.94	6.68
2006	6.38	5.48	5.24	6.76
2007	5.93	5.32	5.55	6.37
2008	6	5.12	5.75	6.28
2009	4.86	4.39	5.2	5.39
2010	6.1	4.56	5.14	4.77
2011	4.73	4.53	5.12	5.01
2012	5.2	4.34	5.03	4.79
2013	5.41	4.23	4.63	5.29
2014	4.43	4.65	4.33	4.73
2015	4.82	4.22	4.63	4.68
2016	4.62	4.2	4.13	4.69
2017	4.41	4.21	4.33	3.69
2018	4.24	4.72	4.36	3.1
2019	4.72	3.35	4.37	3.37
2020	7.55	5.31	5.67	4.5
2021	6.02	6.32	5.99	5.13
2022	5.17	5.34	4.83	4.25
2023	5.63	4.05	4.3	3.63
**Number of Joinpoints (Joinpoint years)**	2 (2019, 2020)	2 (2019, 2021)	2 (2019, 2021)	2 (2019, 2021)
**APC-Segment 1 (95% CI)**	-2.0%* (-3.0 to -1.2)	-2.7%* (-3.5 to -1.9)	-1.9%* (-2.3 to -1.5)	-4.1%* (-5.0 to -3.4)
**APC-Segment 2 (95% CI)**	51.1% (-6.3 to 162.1)	35.7%* (13.0 to 68.4)	15.2%* (2.6 to 45.4)	22.1%* (13.5 to 32.9)
**APC-Segment 3 (95% CI)**	-10.2% (-43.8 to 9.5)	-21.2%* (-35.7 to 6.2)	-16.9%* (-34.1 to -6.4)	-16.9%* (-23.7 to -10.6)

Table S5. Myocarditis-related age-adjusted mortality rate (AAMR) in 1999-2019 and 2020-2023 and change in AAMR at the state level in the United States, 1999-2023.

State	AAMR for 1999-2023	AAMR for 1999-2019	AAMR for 2020-2023	Change in AAMR from 1999-2019 to 2020-2023
**Alabama**	3.50	3.56	3.21	-0.35
**Alaska**	5.79	5.19	8.725	-
**Arizona**	4.58	4.6	4.49	-0.11
**Arkansas**	4.03	3.87	4.83	0.96
**California**	5.62	6.04	3.6	-2.44
**Colorado**	9.04	8.96	9.41	0.45
**Connecticut**	3.55	3.39	4.33	0.94
**Delaware**	4.63	4.53	5.105	-
**District of Columbia**	6.88	7.06	5.995	-
**Florida**	5.32	5.48	4.61	-0.87
**Georgia**	5.84	5.93	5.45	-0.48
**Hawaii**	7.16	6.86	8.58	1.72
**Idaho**	3.66	3.42	4.64	1.22
**Illinois**	3.90	3.93	3.75	-0.18
**Indiana**	3.73	3.67	4.02	0.35
**Iowa**	6.49	6.21	7.87	1.66
**Kansas**	5.85	6.35	3.35	-3
**Kentucky**	3.63	3.71	3.26	-0.45
**Louisiana**	6.04	5.45	9.03	3.58
**Maine**	6.17	6.02	6.92	0.9
**Maryland**	8.57	8.75	7.73	-1.02
**Massachusetts**	4.37	4.38	4.32	-0.06
**Michigan**	4.28	3.98	5.83	1.85
**Minnesota**	5.27	5.27	5.25	-0.02
**Mississippi**	3.58	3.49	4.03	0.54
**Missouri**	3.45	3.16	4.88	1.72
**Montana**	3.54	3.3	4.655	-
**Nebraska**	7.52	6.51	12.36	5.85
**Nevada**	2.90	2.75	3.54	0.79
**New Hampshire**	4.41	4.4	4.47	0.07
**New Jersey**	7.02	7.24	5.95	-1.29
**New Mexico**	4.74	4.98	3.57	-1.41
**New York**	6.06	5.88	6.96	1.08
**North Carolina**	4.55	4.66	4.08	-0.58
**North Dakota**	5.23	4.51	8.61	4.1
**Ohio**	7.39	7.8	5.34	-2.46
**Oklahoma**	3.58	3.25	5.18	1.93

**Oregon**	3.36	3.29	3.67	0.38
**Pennsylvania**	4.80	4.45	6.57	2.12
**Rhode Island**	8.28	8.92	5.09	-3.83
**South Carolina**	9.24	9.59	7.67	-1.92
**South Dakota**	5.47	4.89	8.18	3.29
**Tennessee**	6.70	6.47	7.78	1.31
**Texas**	4.68	4.46	5.63	1.17
**Utah**	7.00	7.62	4.47	-3.15
**Vermont**	4.96	4.74	6.045	-
**Virginia**	3.04	3.1	2.77	-0.33
**Washington**	4.00	3.98	4.08	0.1
**West Virginia**	5.41	5.76	3.56	-
**Wisconsin**	4.74	4.59	5.5	0.91
**Wyoming**	5.76	5.67	6.22	-
**All US**	5.23	5.24	5.16	-0.08

Table S6. Observed and expected myocarditis deaths and contribution of COVID-19 infection to the excess myocarditis deaths

Year	Observed myocarditis-related deaths	Expected myocarditis-related deaths (95% CI)	Excess myocarditis-related deaths (observed deaths – expected deaths)	Excess deaths as a percentage of expected deaths (%)	Proportion of excess deaths/all-myocarditis-related deaths (%)	Myocarditis-related deaths with COVID-19 mentioned as a cause of death	Percentage of excess myocarditis-related deaths with COVID-19 mentioned as a cause of death (%)	Percentage of excess myocarditis-related deaths without COVID-19 mentioned as a cause of death (%)
**2020**	1584	1077 (1026 - 1127)	507	47.1	32.0	367	72.34	27.66
**2021**	1648	1064 (1012 - 1115)	584	54.9	35.5	445	76.15	23.85
**2022**	1394	1036 (982 - 1090)	358	34.6	25.7	271	75.67	24.33
**2023**	1253	1019 (956 - 1083)	234	22.9	18.6	101	43.25	56.75
**2020-2023**	5879	4196 (3976 - 4415)	1683	40.1	28.6	1184	70.33	29.67

Table S7. Sensitivity analysis of most frequently mentioned underlying (primary) causes of death for all myocarditis-related deaths for cumulative 2020-2023, year 2021 and cumulative 2016-2019 for comparison.

**2020-2023**			
**Underlying Cause of death**	**Underlying Cause of death Code**	**Deaths**	**Proportion of all myocarditis related deaths (%)**
**All myocarditis related deaths**	I51.4, I40.x	5879	-
**Myocarditis, unspecified**	I51.4	1483	25.2
**COVID-19**	U07.1	1080	18.4
**Infective myocarditis**	I40.0	385	6.5
**Atherosclerotic heart disease**	I25.1	298	5.1

**Acute myocarditis, unspecified**	I40.9	191	3.2
**Atherosclerotic cardiovascular disease, so described**	I25.0	146	2.5
**Acute myocardial infarction, unspecified**	I21.9	145	2.5
**Cardiomegaly**	I51.7	129	2.2
**Accidental poisoning by and exposure to other and unspecified drugs, medicaments and biological substances**	X44	112	1.9
**2021**			
**Underlying Cause of death**	**Underlying Cause of death Code**	**Deaths**	**Proportion of all myocarditis related deaths (%)**
**All myocarditis related deaths**	I51.4, I40.x	1648	-
**COVID-19**	U07.1	417	25.3
**Myocarditis, unspecified**	I51.4	384	23.3
**Infective myocarditis**	I40.0	84	5.1
**Atherosclerotic heart disease**	I25.1	75	4.6
**Acute myocarditis, unspecified**	I40.9	53	3.2
**Atherosclerotic cardiovascular disease, so described**	I25.0	47	2.9
**Acute myocardial infarction, unspecified**	I21.9	45	2.7
**Cardiomegaly**	I51.7	41	2.5
**Accidental poisoning by and exposure to other and unspecified drugs, medicaments and biological substances**	X44	34	2.1
**Hypertensive heart disease without (congestive) heart failure**	I11.9	23	1.4
**Accidental poisoning by and exposure to narcotics and psychodysleptics [hallucinogens], not elsewhere classified**	X42	20	1.2
**2016-2019 (pre-pandemic)**			
**Underlying Cause of death**	**Underlying Cause of death Code**	**Deaths**	**Proportion of all myocarditis related deaths (%)**
**All myocarditis related deaths**	I51.4, I40.x	4532	-
**Myocarditis, unspecified**	I51.4	1509	33.3
**Infective myocarditis**	I40.0	395	8.7
**Atherosclerotic heart disease**	I25.1	307	6.8
**Acute myocarditis, unspecified**	I40.9	213	4.7
**Cardiomegaly**	I51.7	154	3.4
**Atherosclerotic cardiovascular disease, so described**	I25.0	150	3.3
**Acute myocardial infarction, unspecified**	I21.9	100	2.2
**Accidental poisoning by and exposure to other and unspecified drugs, medicaments and biological substances**	X44	89	2.0
**Hypertensive heart disease without (congestive) heart failure**	I11.9	83	1.8
**Accidental poisoning by and exposure to narcotics and psychodysleptics [hallucinogens], not elsewhere classified**	X42	65	1.4

Table S8. ARIMA model diagnostics and metrics for expected AAMRs and number of deaths.

Model diagnostics and metrics	ARIMA for expected AAMRs	ARIMA for expected number of deaths
ADF test (p-value)	0.34	0.98
Differencing applied	Yes (d = 1)	Yes (d = 1)
Selected ARIMA model (p,d,q)	(2,1,0)	(2,1,0)
BIC	13.15	90.24
Training RMSE	0.0618	20.04
Training MAE	0.0494	16.90
Training MAPE (%)	1.09%	1.43%
ACF1 of residuals	-0.0830	-0.0137
Ljung-Box p-value	0.4753	0.6387
Rolling cross-validation RMSE	0.5923	96.88

Table S9. Forecast of expected AAMRs from 2020 to 2023 using the ARIMA model.

Year	AAMR (95% CI)
2020	3.82 [3.66 - 3.98]
2021	3.76 [3.60 - 3.92]
2022	3.62 [3.45 - 3.78]
2023	3.48 [3.29 - 3.68]

Supplement Figure S1. Sensitivity analysis for Myocarditis-related AAMR from multiple cause-of-death data (all myocarditis-related deaths) and the underlying cause of death data.

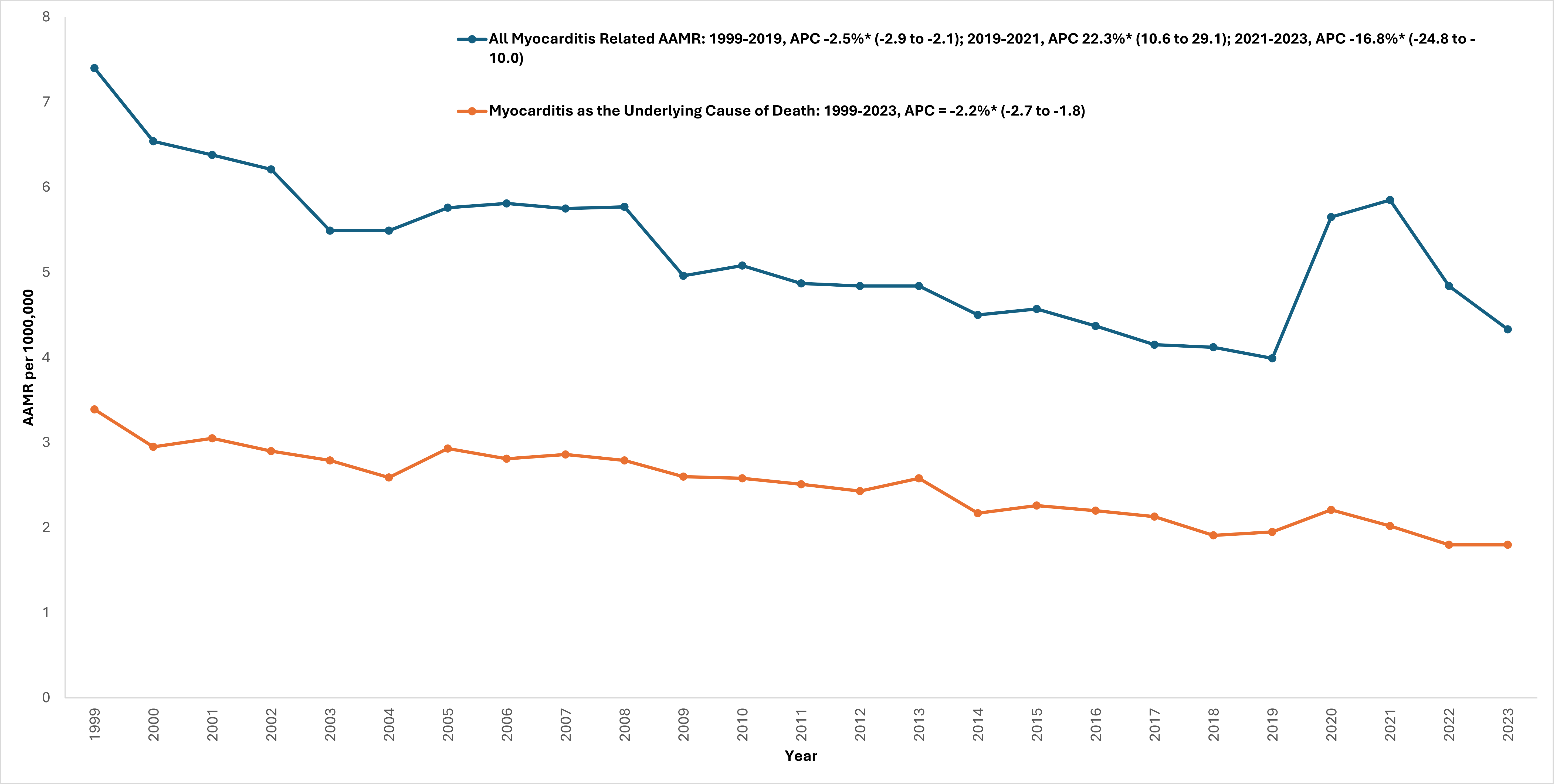

Supplement Figure S2. State-level myocarditis-related AAMR from 1999 to 2019 and 2020 to 2023

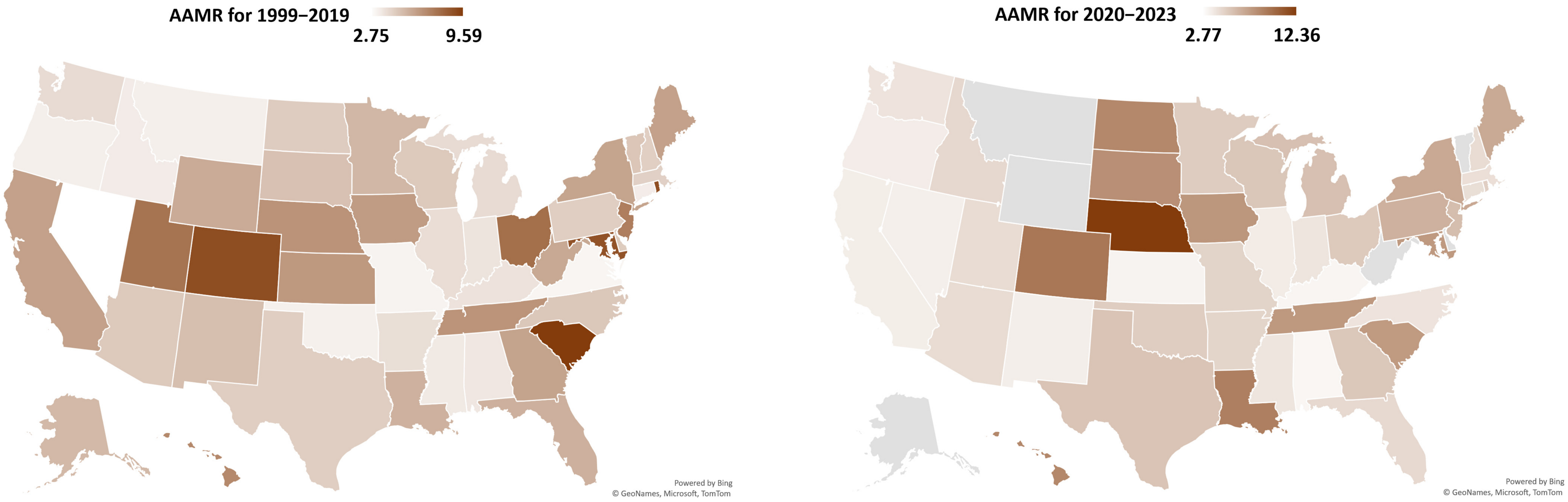

Supplement Figure S3a. State-level change in Myocarditis-related age-adjusted mortality rates (AAMR) in the United States from 1999-2019 to 2020-2023

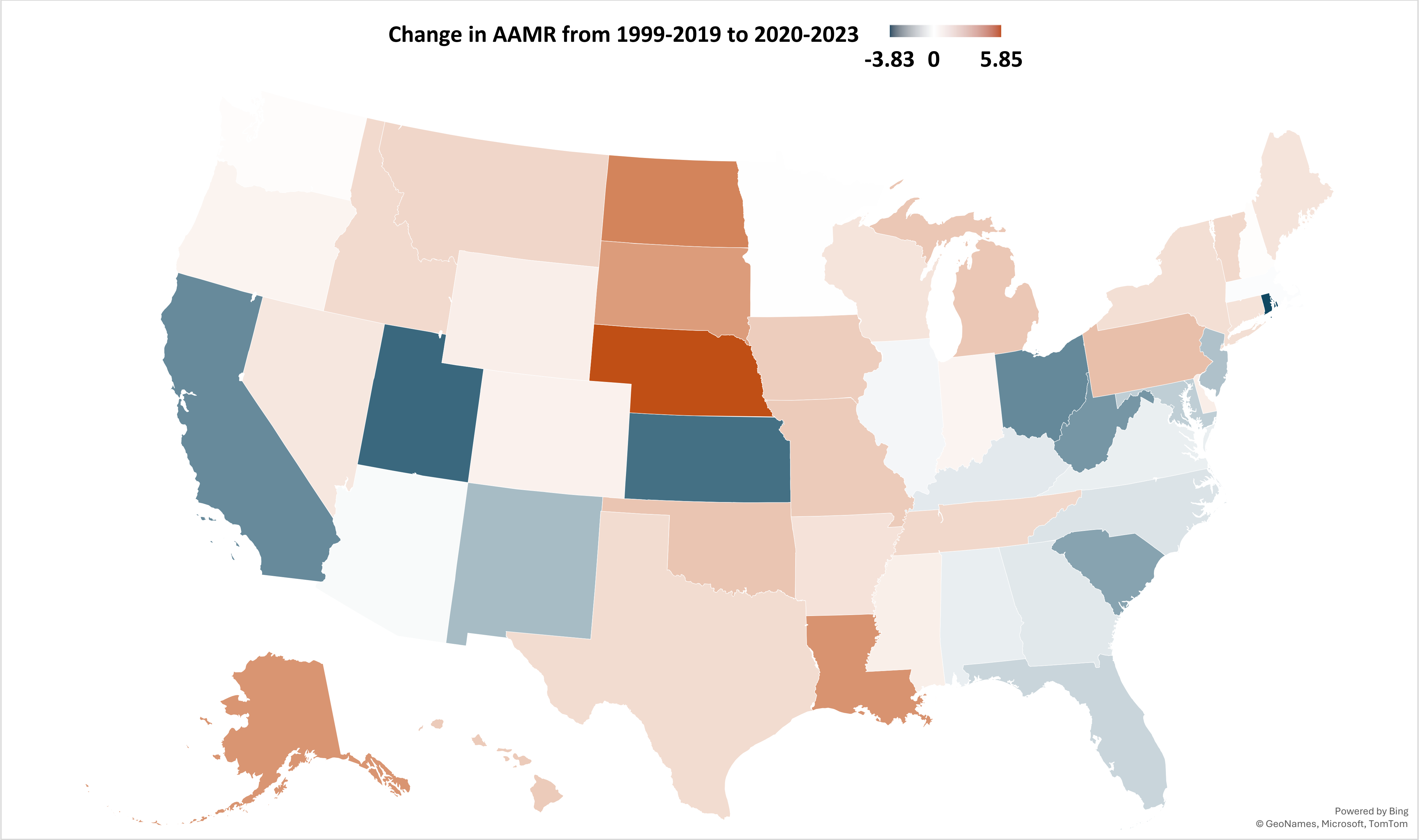

Supplement Figure S3b. State-level change in Myocarditis-related age-adjusted mortality rates (AAMR) in the United States from 1999-2019 to 2020-2023

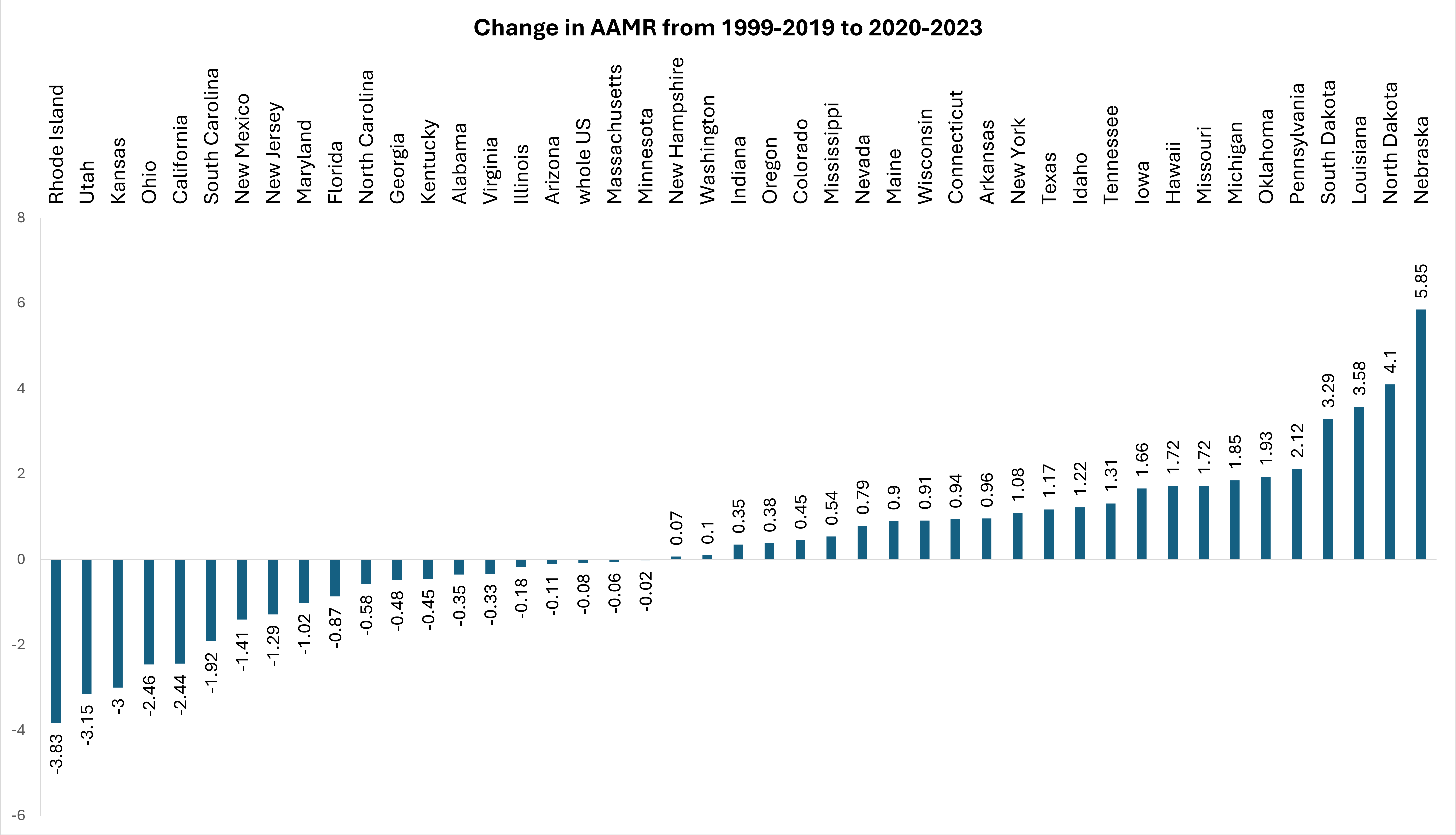

Supplement: Supplementary file 1 [file jcm-14-05116-s001.zip › jcm-3733833-supplementary.pdf]
